# How often are medication prescribed in the emergency appointment at Egas Moniz Dental University Clinic? – a pilot study

**DOI:** 10.1080/07853890.2021.1897347

**Published:** 2021-09-28

**Authors:** Inês Mocho, Ana Filipa Rosário, Luís Santos, Eduardo Guerreiro, Ana Delgado, José João Mendes

**Affiliations:** a Instituto Universitário Egas Moniz (IUEM), Egas Moniz Cooperativa de Ensino Superior, Caparica, Portugal; b Egas Moniz Dental Clinic (EMDC), Egas Moniz Cooperativa de Ensino Superior, Caparica, Portugal; c Clinical Research Unit (CRU), Egas Moniz Cooperativa de Ensino Superior, Caparica, Portugal

## Abstract

**Introduction:**

In the oral cavity, there are usually several pathogenic microorganisms capable of forming oral/dental infections and subsequent systemic infections [1]. The main reason for the patients to seek medical-dental care is usually condition where pain is always present [2]. Medication like pain killers and antibiotics are frequently used [1]. Prescription is a personalised act, where it is necessary to define a diagnosis, specify the therapeutic objective, consider the different options and finally choose an effective and safe treatment. Currently, the most common prescribed medication in dentistry are antibiotics and non-steroidal anti-inflammatory drugs (NSAIDs) [3,4].This study aimed to answer the following questions: How often are medication prescribed in the emergency appointment at Egas Moniz Dental University Clinic and what kind of medication are prescribed? This study follows the principles of the Declaration of Helsinki.

**Materials and Methods:**

Pilot Study Observational and Retrospective. Of the 388 emergency appointments, carried out between 1 March 2019 and 31 March 2019 at Egas Moniz Dental University Clinic 249 cases were randomly selected and analysed. Inclusion criteria were all patients who were prescribed medication. Frequency tables were performed according to gender, age and the type of drug prescribed (antibiotic, analgesic and/or NSAIDs).

**Results:**

In 249 emergency appointments, only 50 were prescribed medication. The most common prescribed were antibiotics (*n* = 33), followed by NSAIDs (*n* = 30), analgesics (*n* = 10) and others (*n* = 2). The most common antibiotic prescribed was amoxicillin + clavulanic acid − 875 mg + 125 mg (*n* = 17), followed by amoxicillin − 1 g (*n* = 11).

**Discussion and conclusions:**

Medication by itself is not as successful as the dentist local intervention treatment procedure [3]. Studies show that the most prescribed medication in dentistry are antibiotics and NSAIDs [3,4]. Our results confirm that idea. According to our data, the prevalence of prescription in emergency appointments is approximately 20%. Furthermore, it is important to have a rational prescription in order to reduce the disease duration, systemic repercussions and also the appearance of resistance and high costs [5,6]. Future research should include a bigger sample and a longer period.
